# Supplementary figures and images for: Virus-host interactions predictor (VHIP): Machine learning approach to resolve microbial virus-host interaction networks
Source: PLoS Comput Biol. 2024 Sep 18;20(9):e1011649. doi: 10.1371/journal.pcbi.1011649 (PMC11441702; doi:10.1371/journal.pcbi.1011649)

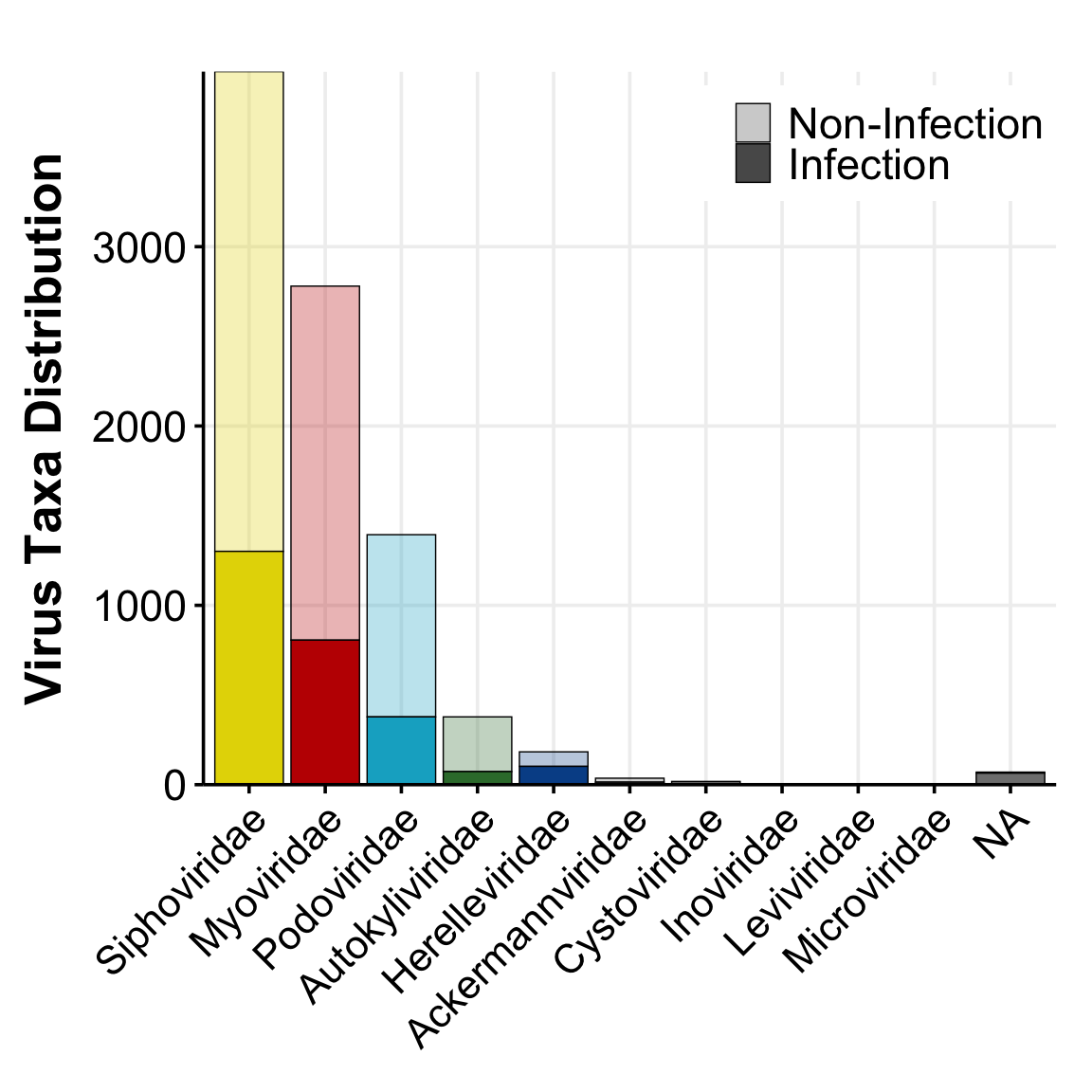

Supplement: S1 Fig — Lighter transparency represents the proportion of non-infection reports by viral family, relative to the solid portion, which represents known infection reports by viral family. (TIF) [file pcbi.1011649.s001.tif]

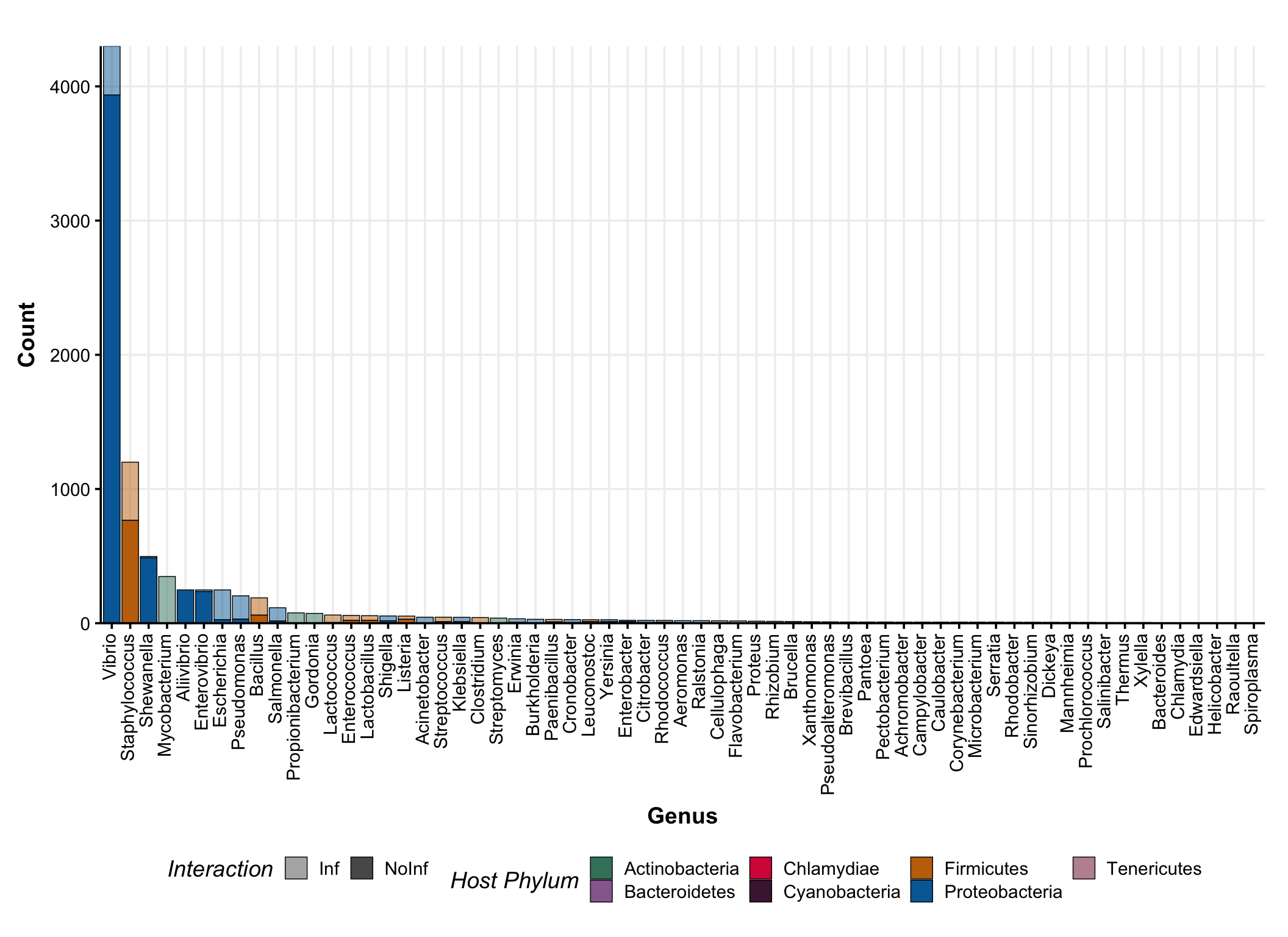

Supplement: S2 Fig — Lighter color transparency represents the proportion of non-infection relative to infection (solid color). (TIF) [file pcbi.1011649.s002.tif]

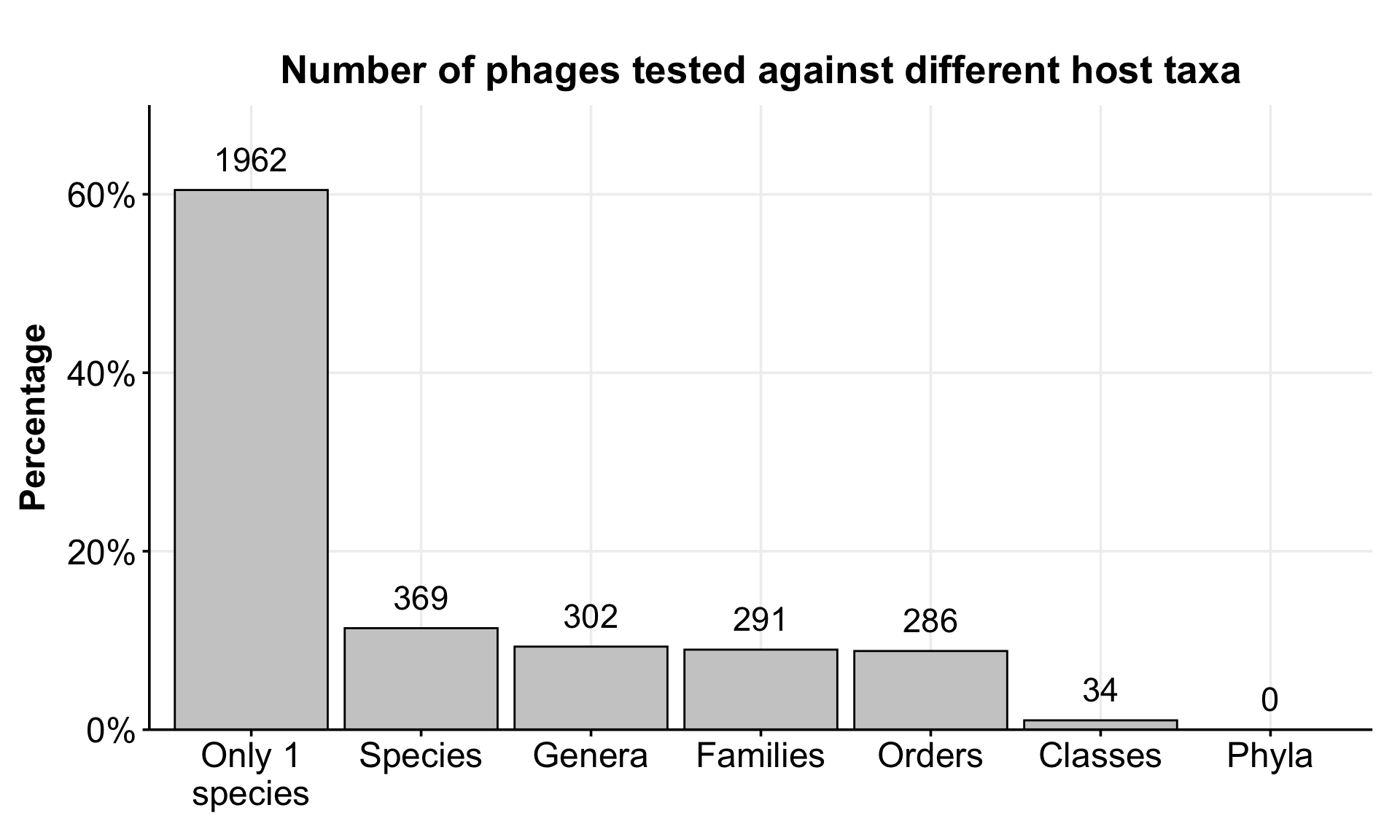

Supplement: S3 Fig — X-axis represent the number of viruses that have been tested. (TIF) [file pcbi.1011649.s003.tif]

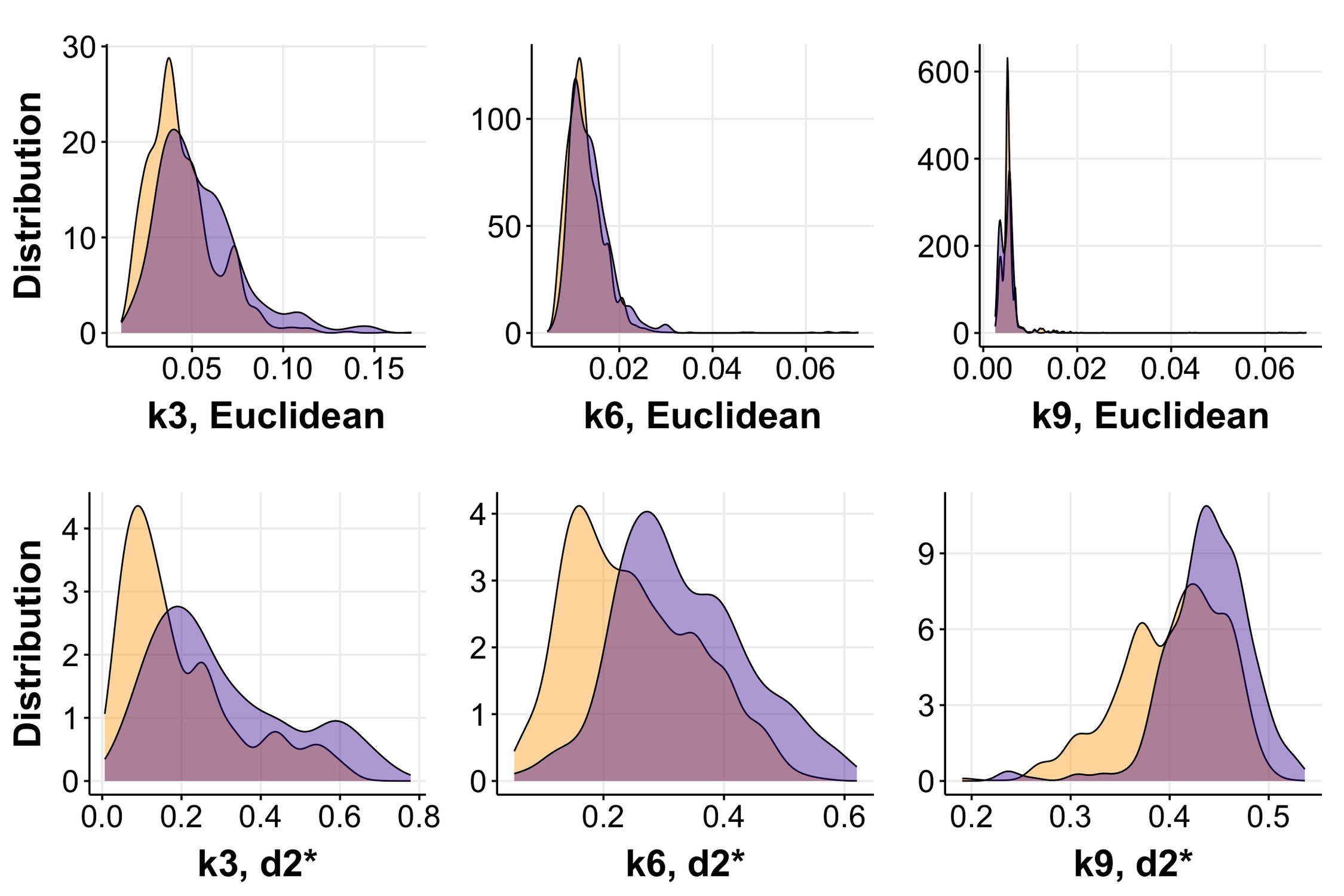

Supplement: S4 Fig — The top row used the Euclidean distance to compute the similarity between the k-mer profiles of the virus and its host, while the second row uses the d2* distance metric. Each column represents a different length of k-mer used to create the k-mer profiles (k-length of 3 versus 6 versus 9). The d2* distance metric is a more appropriate metric than the Euclidean distance metric for the purpose of virus-host prediction since it encodes some evolutionary signals (the peaks for the no-infection and infection are separated). (TIF) [file pcbi.1011649.s004.tif]

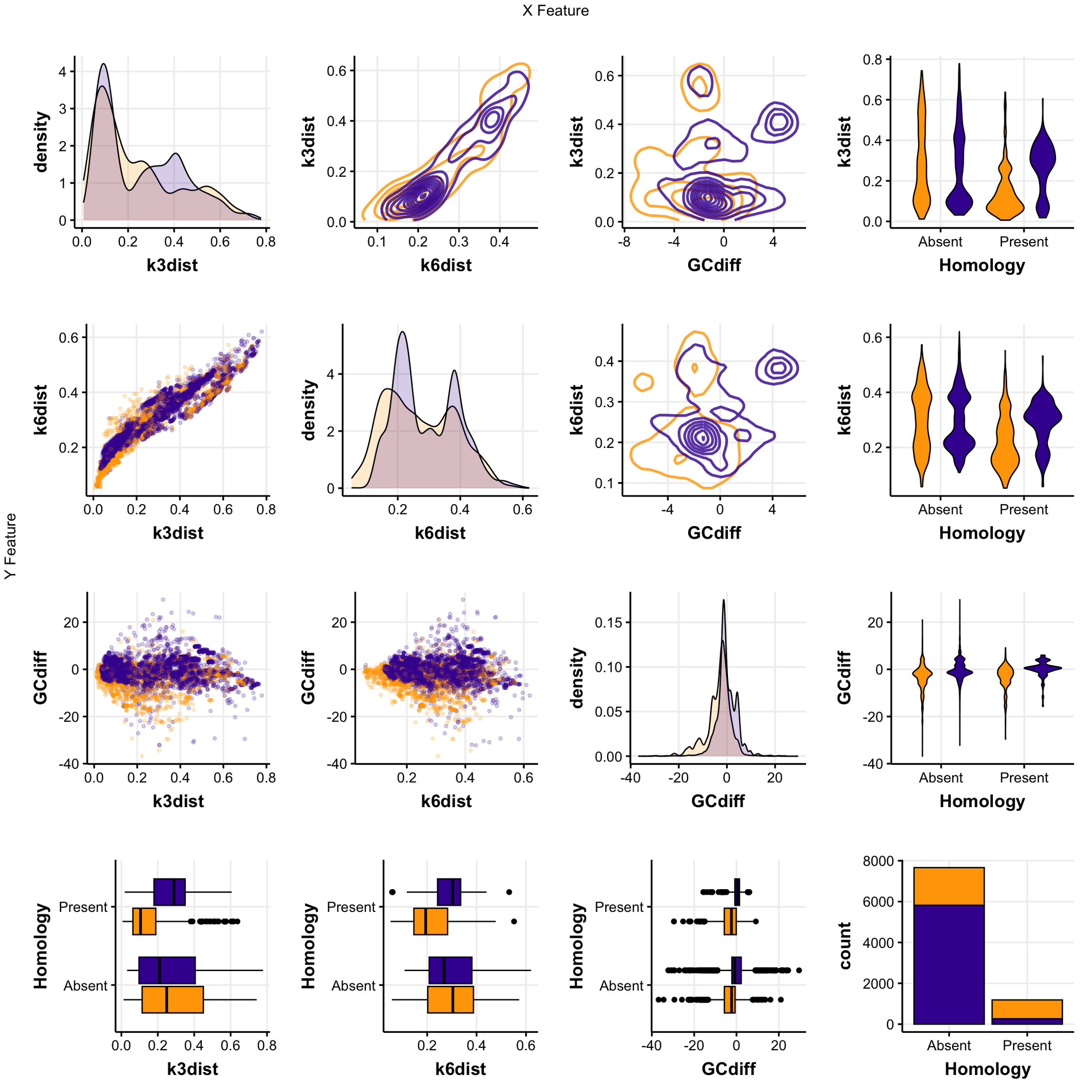

Supplement: S5 Fig — (TIF) [file pcbi.1011649.s005.tif]

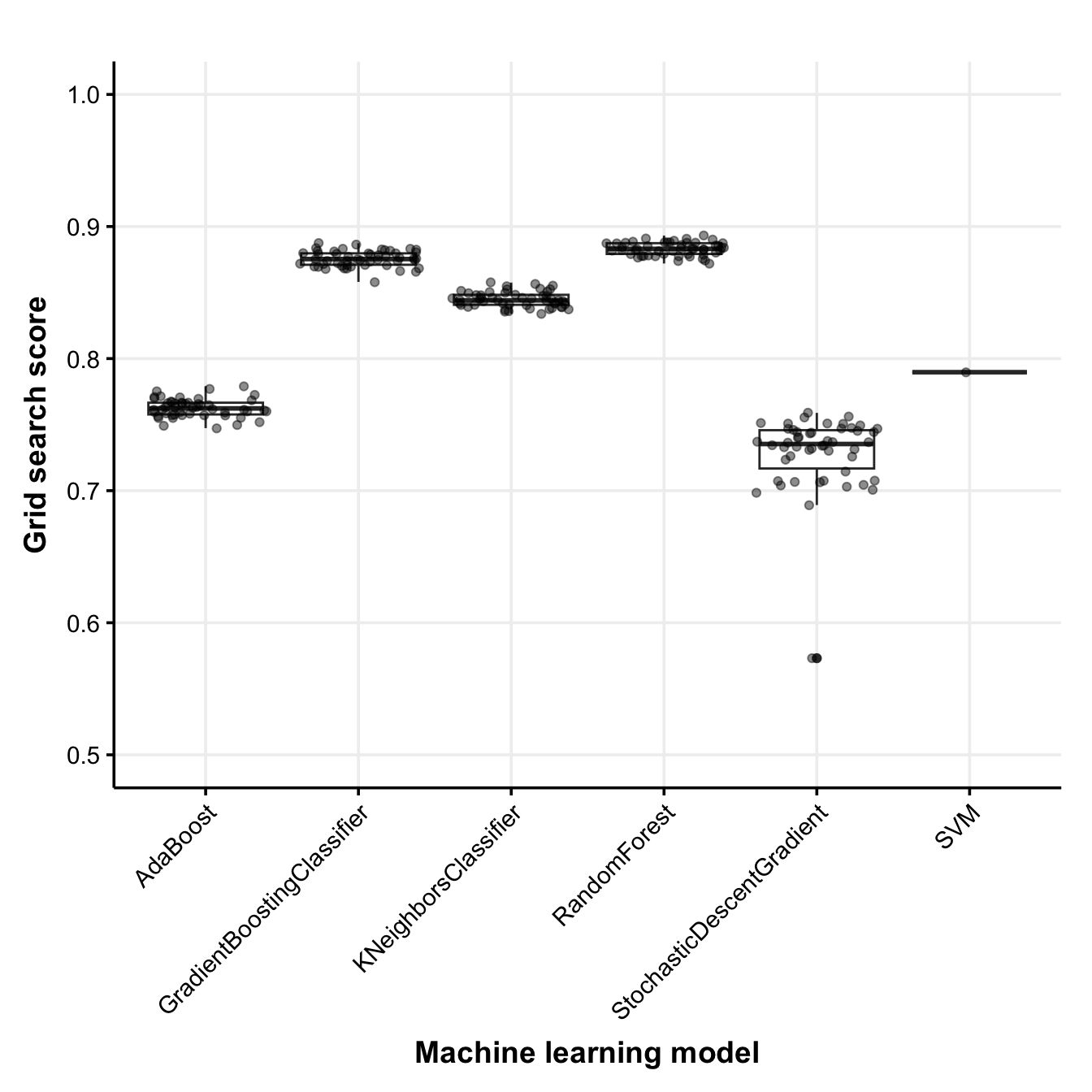

Supplement: S6 Fig — For each type of machine learning model, a grid search was performed to determine the best combinations of parameters. This plot shows the accuracy of the best performing model. This was bootstrapped 50 times (except for SVM since the fit algorithm is O(n^2)). (TIF) [file pcbi.1011649.s006.tif]

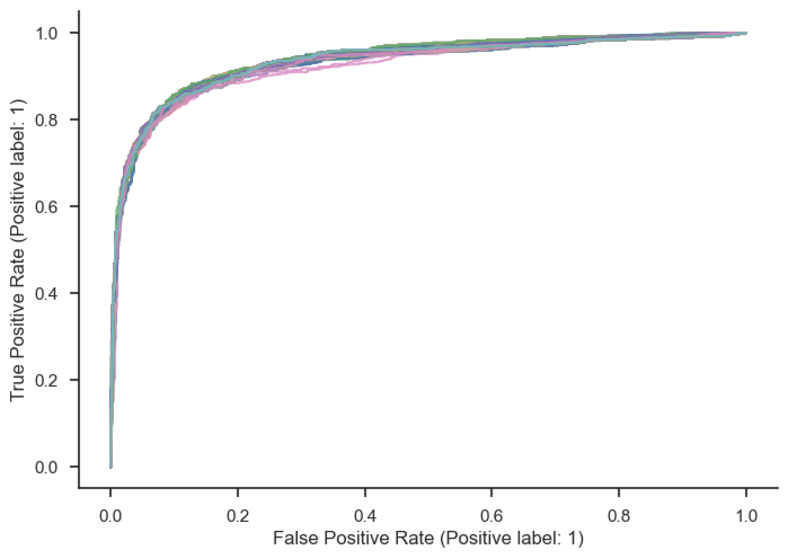

Supplement: S7 Fig — (TIF) [file pcbi.1011649.s007.tif]

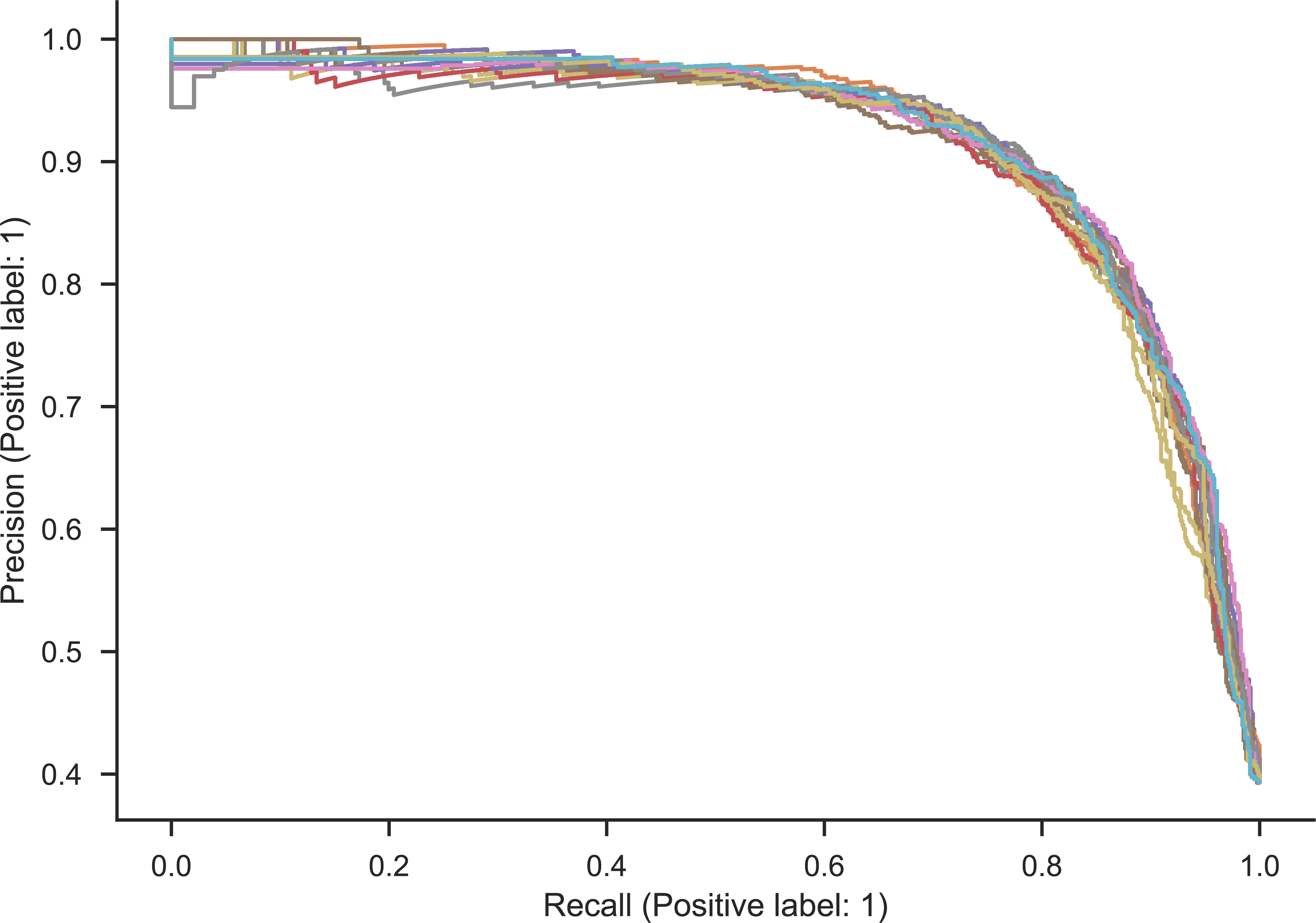

Supplement: S8 Fig — (TIF) [file pcbi.1011649.s008.tif]
