# Supplementary material for: Virus-host interactions predictor (VHIP): Machine learning approach to resolve microbial virus-host interaction networks
Source: PLoS Comput Biol. 2024 Sep 18;20(9):e1011649. doi: 10.1371/journal.pcbi.1011649 (PMC11441702; doi:10.1371/journal.pcbi.1011649)
Supplement: Attachment — Submitted filename: response_to_reviewers.pdf [file pcbi.1011649.s014.pdf]

Response to reviewers

Dear Dr. Duhaime,

Thank you very much for submitting your manuscript "Virus-Host Interactions Predictor (VHIP): machine learning approach to resolve microbial virus-host interaction networks" for consideration at PLOS Computational Biology.

As with all papers reviewed by the journal, your manuscript was reviewed by members of the editorial board and by several independent reviewers. In light of the reviews (below this email), we would like to invite the resubmission of a significantly-revised version that takes into account the reviewers' comments.

In this work, the authors construct a dataset of viral host ranges by combining information from several publicly available sources. Using these data, they evaluate the performance of several existing tools for predicting viral/host interactions based on sequence data and develop a novel method that leverages both sequence and other metadata. The reviewers all highlighted the potential utility of the data set, which I agree with, but also identified several areas where substantive additional work is needed.

First, the reviewers all agreed that the biased nature of the host/viral data wrt to host limited the impact of both the new dataset and our interpretation of the novel machine learning method. My suggestion is that the authors demonstrate more rigorously how model performance varies when considering the smaller number of non-human pathogenic viruses included in the study. For example, the authors could split training/testing data between human pathogenic and all other viruses or could do a kind of repeated bootstrap or downsampling such that human pathogenic vs. non-human pathogenic sample sizes are equal.

Additionally, the authors should further discuss what might be different wrt to model performance, etc. if more diverse data sets were available.

Second, the reviewers point out that while a comparison is done between existing classification methods, no comparison seems to have been done between the new developed method and established approaches.

Given how much emphasis in the paper is placed on both the specific new method and, more interestingly, the general approach of including more metadata in the prediction models, the Importantly, the authors must also engage data set bias when comparing across models. I can imagine a number of pernicious kinds of bias that could show up here related to metadata coverage in human vs. non-human pathogenic viruses, etc.

Third, a number of claims in the paper, e.g., the network results in F6, seem to go well beyond what is demonstrated in the paper. My advice here is to carefully weigh what is and isn't a well supported conclusion in the current study. For example, given the heavy reliance on two data

sets, it does seem unlikely that much can be said by generalizability across environments.
I hope that the authors pay careful attention to the detailed reviewer comments and understand that addressing them will almost certainly require running new analysis and a meaningful re-write of aspects of the manuscript.

We cannot make any decision about publication until we have seen the revised manuscript and your response to the reviewers' comments. Your revised manuscript is also likely to be sent to reviewers for further evaluation.

When you are ready to resubmit, please upload the following:

[1] A letter containing a detailed list of your responses to the review comments and a description of the changes you have made in the manuscript. Please note while forming your response, if your article is accepted, you may have the opportunity to make the peer review history publicly available. The record will include editor decision letters (with reviews) and your responses to reviewer comments. If eligible, we will contact you to opt in or out.

[2] Two versions of the revised manuscript: one with either highlights or tracked changes denoting where the text has been changed; the other a clean version (uploaded as the manuscript file).

Important additional instructions are given below your reviewer comments.

Please prepare and submit your revised manuscript within 60 days. If you anticipate any delay, please let us know the expected resubmission date by replying to this email. Please note that revised manuscripts received after the 60-day due date may require evaluation and peer review similar to newly submitted manuscripts.

Thank you again for your submission. We hope that our editorial process has been constructive so far, and we welcome your feedback at any time. Please don't hesitate to contact us if you have any questions or comments.

Sincerely,

Samuel V. Scarpino
Academic Editor
PLOS Computational Biology

James O'Dwyer
Section Editor
PLOS Computational Biology

****

Thank you very much for taking the time and effort to review our manuscript. We addressed all comments to the best of our abilities and we hope it meets your expectations.

Before addressing each reviewer's individual comments, we would like to clarify a few high-level recurring themes that emerged in the reviews and that we took care to address.

First, there were frequent references to "human pathogenic viruses and non-human pathogenic viruses," and the potential biases in model performance that this dichotomy posed. However, there were no human viruses included in this study; this work considers only viruses that infect bacteria and archaea. As such, we revised the text to more explicitly clarify this point early in the manuscript, but could not directly address comments regarding comparing the performance of VHIP on human viruses and other eukaryotic viruses. We changed the wording from host to microbe to emphasize that only viruses that can infect bacteria or archaea were considered. In addition, the metadata describing virus-host pairs (data source, taxonomic information) were not considered as predictive features. This is because the fundamental motivation for the development of VHIP is to predict virus-host interactions for a given query of unknown, uncharacterized viruses and hosts of interest from genomic sequences (e.g., such as might be reconstructed from de novo metagenome assemblies).

Second, there were many comments regarding comparing the accuracy of VHIP to existing tools. Unfortunately, a one-to-one comparison between VHIP and existing tools is not possible for multiple reasons—in fact this challenge embodies the novelty of the VHIP approach and the need it meets in the research community. First, existing tools only take viruses as input (not putative host genomes), and test against a predefined database of hosts. As such, if there is a host that is not included in the database, those tools are not able to predict that host. More importantly, what renders a meaningful comparison between existing tools and VHIP impossible is the differing definitions of accuracy (which derive from each tool's design, algorithm, and end goals). Accuracy for VHIP is determined by its ability to predict either infection or non-infection for any given virus-host pair. In other words, it is the True Positive rate + the True Negative rate. For existing tools, they define accuracy by only considering their best predicted host. Here is an excerpt from the most recent tool published, IPHoP: "For all the tools, the best prediction was taken for each input virus based on the relevant metric and considered as correct if the genus of the predicted host genome or the predicted genus for tools predicting host taxonomy was consistent with the information collected from the reference database". As such, their definition of accuracy focuses on predicting at least one correct host. If a virus is able to infect multiple different species, those tools only have to predict a single one of them (as their best prediction) to get 100% accuracy. If a virus is able to infect 5 different species, the fact that those tools are missing 4 of them is not taken into account. They predict taxa, and as long as one of the known hosts for a given virus is a member of the predicted taxa, the model is considered to be accurate. This also leads to spurious interpretation. If a virus is predicted to infect a genus, does it mean this virus is able to infect all species belonging to that genus? A few? Or just one? And if just one, which one? This issue with existing tools is what drove us to design a tool that focuses on predicting virus-host *interactions* in an "all against all" evaluation. We haven't found a metric that would accurately reflect the accuracy between existing tools and VHIP. We responded to the reviewers to the best of our abilities regarding this specific issue, we added an analysis

comparing how VHIP and existing tools perform in recovering known host range and expanded the discussion regarding the difficulty in comparing existing tools to VHIP in the manuscript (paragraph starting at line 372).

Regarding the final figure, it is not a result from the research presented in this manuscript but an outlook for future research that is made possible by considering many viruses to many hosts interactions. The tool developed presented here is only one of a number of new approaches that are expanding viral research and allowing this type of prediction. Technological advances in sequencing, such as Hi-C, are also allowing this type of analysis. We are currently applying VHIP on new exciting data to resolve such virus-host interactions networks. This work, however, cannot be easily included in this manuscript as it will easily double the length of the manuscript. We are learning that the application of VHIP to real data takes a considerable amount of ground truthing and benchmarking to demonstrate the predicted networks are trustworthy and reliable, which we are excited to demonstrate, but we feel it is beyond the scope of the current manuscript. Our intention with the final figure and section is to share the mechanics of the prediction method (panel A), as well as expand the conversation in the literature about the importance and usefulness of the type of “all against all” networks that this work and others are paving the way for. We believe retaining the final figure and discussion elevates these ideas and has the potential to increase the impact of this publication.

Reviewer #1: I do not have any major issues with the manuscript.

As a suggestion, please consider testing the tool on genomes derived from metagenomes. In particular ones with organisms that are taxonomically distanced from the ones dominating your existing host range datasets. There are some metagenomic datasets that include both long read data and Hi-C to confirm. I believe https://doi.org/10.1016/j.jhazmat.2023.131944 - which you cite - has some. My main concern is the relatively narrow host range your tool was developed on. Will the model generalize?

Thank you for your input! We are very interested in comparing predictions by VHIP with results from Hi-C. The inputs for VHIP are the sequences for the viruses and MAGs for hosts of interest. We found that papers that use Hi-C rarely make both the virus contigs/bins and hosts MAGs available. The study suggested here is unfortunately not an exception, as only the raw sequences (not population genomes/assemblies/MAGs/bins) were made available. In theory, we could reconstruct the virus contigs and host MAGs but due to many stochastic algorithms involved in the metagenomic pipeline, it is near impossible to reconstruct the exact same sequences achieved in that study, rendering a direct comparison between VHIP and Hi-C potentially spurious. However, we have new in house datasets of our own for that in hand and this comparison is included in future evaluation of both Hi-C (which itself is not the gold standard of certainty, it is suspected to both over- and under-estimate virus-host linkages) and VHIP.

Regarding if the model is going to be able to generalize, we need to consider the underlying assumption behind VHIP, which is that virus-host signals of coevolution are equivalent

regardless of host and/or virus taxonomy, and of environmental conditions. If that assumption is violated, then one must be cautious in interpreting the predictions from the model. We are currently exploring applications of VHIP on metagenomics data. Application on real data takes a considerable amount of ground truthing and benchmarking to demonstrate the predicted networks are trustworthy and reliable and we are excited to demonstrate this, stay tuned, but this is beyond the scope of this manuscript.

We expanded the discussion section (paragraph starting at line 431), addressing assumptions regarding the model's ability to generalize. Thank you for your input!

Also, for future work, consider using graph neural networks once you get to ecosystems.

Thank you!

Reviewer #2: In this study, Bastien et al. manually curated a database of virus-host relationship based on publicly available experimental data. Both infection and non-infection information, as well as many-to-many infectious relationships were included, which is distinguishable from many known virus-host databases. Based on this database, the accuracies of commonly used host prediction tolls were validated. The authors then evaluated genomic features between viruses and hosts resulted from coevolution, and based on selected features, they developed a machine-learning based classifier to predict virus-host infectious features based on their genome sequences.

The concept of including non-infection information to host prediction, the attempt in resolving all the infection/non-infection relationships in a community of viruses and hosts as emphasized by the authors are indeed very important. However, they have not clearly demonstrated that the prediction tool they developed has benefitted from these additional data in performance. Moreover, the authors need to provide a user-friendly implementation of this tool online.

Thank you for your feedback! Regarding the inclusion of non-infection data points, it is both an empirical observation and a predicted class of interaction that existing models ignore. Thus, the inclusion of non-infection serves two purposes: 1) it allows the model to learn from genomic-based signals of virus-host coevolution without making any assumptions regarding virus host range (existing models assume that viruses are specialists and have narrow host range to quickly and easily generate negative data), and 2) it also allows to train a model that can predict both infection and non-infection interactions. The latter is especially novel as existing models that use genomic sequences for predictions can only predict infection.

We added a sentence emphasizing the benefits of including non-infection data: "Inclusion of infection and non-infection data points in VHRnet allows us to train a model predicting either infection or non-infection without any assumptions about virus host range." (line 297). To demonstrate that VHIP benefited from the inclusion of non-infection data points, we added a

new analysis comparing VHIP and existing tools' ability to predict known host range (paragraph starting at line 372).

The tool has now been made available on both PyPI and conda-forge. We extensively overhauled the Github repository to include an easy to run example that any interested user can run to check the installation was successful.

Major concerns.

(1) As the authors have admitted, the VHRnet database is heavily biased to human pathogens, phage therapy studies, and the data from the two experimental studies they utilized. There are so many potential relationships between the hosts and viruses in the database are not yet tested by experiments. Therefore, its current version can provide little valuable information to assess the topic of host specificity of viruses. Please move lines 164 to 175 to the Discussion, and instead of indicating that your database indicates that viruses are specialists, you should point out that the VHRnet database currently may provide little valuable information to host specificity of viruses.

We are glad we can clarify this. The VHRnet dataset contains viruses infecting bacteria and archaea, but contains no information of viruses infecting eukaryotic organisms. However, it is heavily biased towards viruses infecting microbial hosts that are of concern to human health (i.e., *Staphylococcus aureus*).

We also agree with the reviewer that there are so many potential relationships between the hosts and viruses that are not yet tested by experiments, which is highlighted by the sparsity of the network presented in figure 2A. If everything was tested against everything, then there would be edges connecting all viruses to all hosts in the network. We argue that because there are so many missing edges, one must be careful when talking about the specificity of a given virus. We do not think that the dataset indicates that viruses are specialists, but it does highlight both the lack and need of testing more diverse virus-host pairs.

We moved the requested lines to discussion and expanded discussion regarding assessing host specificity of viruses (paragraph starting at line 398).

(2) The authors explained why they did not compare the performance of their tool to other published host prediction tools with a very vague description. They seem to say that VHIP is designed for predict virus-host pair combination while all others predict hosts based on highest scores? I am not convinced. I think you can certainly compare the accuracy of VHIP with others for any single host-virus pairs as you have done for the existing tools in Fig. 3.

That is correct. Existing tools take input viral sequences and compare the signal of coevolution against a predefined host database. The output is a list of potential hosts with varying scores. When those tools assess their predicting power, they only consider the taxa of the host with the top score.

Here is an excerpt from the most recent tool published, IPHoP (emphasis is ours): “For all the tools, **the best prediction** was taken for each input virus based on the relevant metric and considered as **correct if the genus of the predicted host genome or the predicted genus for tools predicting host taxonomy was consistent with the information collected from the reference database**”.

To further illustrate the issue between their approach and our approach: let’s imagine a virus that infect species A and species B, and those species belong to different genus. For existing tools, they will obtain 100% accuracy as long as their top score includes either species A or species B. For VHIP, because it considers non-infection prediction, it will only obtain 100% accuracy if it can predict infection for both species. This problem gets exacerbated for existing tools when considering viruses that have broader host ranges, for which there are an increasing number of “right answers” to pick from resulting in an inflated and spurious accuracy. This is the fundamental issue we encountered when attempting comparing VHIP to existing tools.

To address the comment regarding the vague description, we expanded our discussion regarding the challenge of comparing VHIP to HPTs in line 372. In addition, we included a new SI table containing the predictions of existing tools on viruses present in the VHRnet dataset, and we hope this helps to clarify questions regarding how existing tools function.

Finally, in an attempt to enable comparison and demonstrate how VHIP performs better than HPTs, we challenged HPTs against known virus-host pairs and recorded their top predictions. Since HPTs were trained on virus-host pairs extracted from the virus NCBI database, we used from the Nahant Collection and Staphylococcus study that was not used during training of VHIP, ensuring novel data points across all tools. For each virus-host pair evaluation, we queried VHIP’s infection prediction and checked if the correct host was included in each HPT’s output. We added this new analysis in the results section at line 377.

The data from Figure 3 was used as part of the training and testing set needed for VHIP, which particularly needs true negative data.

The authors reported an overall accuracy value of 87.8%, but it is not explained how this value is obtained? I can not found relevant figures or tables for the results of this calculation.

Thank you for pointing out this oversight. The accuracy of the tool is defined as the number of correct predictions divided by the number of total predictions on the test set when training the final model using best hyperparameters. We made the definition of accuracy used in this manuscript clearer in both the results (line 336) and method sections (line 587).

(3) I checked the github website of VHIP, but it looks like that VHIP is not yet fully implemented in a user-friendly way. The pretreatments for data needs to be done by the users with additional tools and procedures, which lacks of necessary details in introduction. This exclude most of the users without rich experience in bioinformatics. Please fully implement VHIP as a conda

package or at least easy to use. Also please provide a detailed instruction for installation and use of this software.

Thank you for the feedback! We expanded the documentation on the Github repository, included an easy to run example to check installation, and the tool is now made available on both PyPI and conda-forge.

(4) The discussion of virus-host networking modeling is way beyond the currently results can infer. Please delete Fig. 6 and most of the relevant discussion, unless you can provide the results showing that you have applied VHIP on a specific environmental data to build and explore a network. Instead, please discuss more about the accuracy and reliability of your tool.

Thank you for your insightful comment and feedback! Figure 6 is not a result from this study, but an outlook of future research that is enabled by considering many viruses to many hosts interactions. We are currently applying VHIP on new exciting data to resolve such virus-host interactions networks. This work, however, cannot be easily included in this manuscript as it will easily double the length of the manuscript. We are learning that the application of VHIP to real data takes a considerable amount of ground truthing and benchmarking to demonstrate the predicted networks are trustworthy and reliable, which we are excited to demonstrate, but we feel it is well beyond the scope of the current manuscript. Our intention here is to share the mechanics of the prediction method, as well as expanding the conversation in the literature about the importance and usefulness of networks that this work and others are paving the way for.

We expanded the discussion regarding the accuracy and reliability of the tool as suggested (paragraph started at line 363 in the result section and at line 431 in the discussion section).

Minor comments.

Line 132. Legends of C and D should be swapped.

Done.

Line 235. Methods to identify HGT is not found, please specify the sequence identity threshold here.

Thank you for identifying this oversight. This has been fixed and reflected in the manuscript in the results section at line 221 and in the method section at line 536.

Line 364. a stronger signal by compared to what?

Thank you for pointing this out! We clarified the sentence: “Interestingly, all the features used by the model contain information that can be leveraged for predictions, but based on feature

importance determined by the model during the training phase, sequence composition features are the most useful for virus-host predictions (Fig 5D), with %G+C difference encoding the strongest signal the model leverages" (line 354)

Fig 5D. Please order by the one with highest importance on the top and the ones with lower importance following up.

Done.

Reviewer #3: Overall thoughts

This paper trains a Gradient Boosted Machine to predict the presence of a successful interaction between viruses and bacterial hosts using genome features as predcitors. While the creation of these genome features is outside of my immediate expertise, I found they employed a nice use of CRISPR motifs in host and virus genomes as a signal of past co-evolution, even though they found that these motifs were rare in their data. The authors conclude that features including GC differences and k-mer sequence motifs were the most useful features.

While I think this paper is an excellent forway into the use of both virus and host genome features to predict virus-host interactions, I found that the paper largely over-sold it's novelty in terms of methodology, and the production of a new and valuable dataset of experimentally verified infections. In particular, the authors claim novelty of this paradigm of binary network prediction for species interactions, which indicates they did not conduct a thorough literature review. Further, their dataset is largely an amalgamation of two existing datasets, which are narrow either taxnomically or sample a single environment. They do add new virus-host interactions to these published records, but they are mined from GenBank metadata without any discussion of validation of these records, or why they assume them to be controlled experimental infections as opposed to naturally infected hosts. From a methods perspective, despite acknowledging their limited and unbalanced training data, the authors do not explore any methods for balanced sampling / re-sampling / data augmentation to help these imbalance issues.

Finally, while the authors provide a nice general discussion, they do not provide any ecological or evolutionary context for the specific predictions made by their model. This would greatly improve the general interest of the work, and also help to open the "black box" of their model.

I'm sorry I cannot be more positive. I think this has the potential to be a very good paper if more attention is paid to the data and modelling, and the claims of novelty and applicability are reduced, and the model and results are placed in a better context with respect to the authors study system (bacteriophages), and the broader landscape of species interaction models.

Major comments

Insufficient review of literature and overall context for the current work:

Lines 40-43 & 82-93: There are multiple virus-host prediction models that predict muliple interactions at once (e.g. the entire network or parts of the sub-network). Further, many existing models successfully reconstuct multiple features of these networks, and in many cases the accuracy of these models excees that reported in your abstract (87.8%) From these statements and the cited references, it is clear that the authors have not sufficiently reviewed existing literature / methods in this field. For example, relevant literature includes:

- Albery et al. (2021) The Science of the host-virus network. Nature Microbiology
- Wardeh et al. (2021) Divide-and-conquer: machine-learning integrates mammalian and viral traits with network features to predict virus-mammal associations. Nature Communications
- Farrell et al. (2022) Predicting missing links in global host–parasite networks. Journal of Animal Ecology
- Elmasri et al. (2020) A hierarchical Bayesian model for predicting ecological interactions using scaled evolutionary relationships. Annals of Applied Statistics
- Poisot et al. (2023) Network embedding unveils the hidden interactions in the mammalian virome. Patterns
- Strydom et al. (2023) Graph embedding and transfer learning can help predict potential species interaction networks despite data limitations. Methods in Ecology & Evolution

I strongly suggest the authors conduct a mor thorough review of alternative approaches for host-pathogen prediction, and adjust their claims accordingly. In particular, in Figure 6 you say your predictions can be visualized as a bipartite network of all virus-host interactions. There is a wealth of information and models on bipartite network models which could help to inform your approach.

Thank you for your comment and sharing this large body of work papers. We changed our wording to acknowledge the focus of studying many viruses to many hosts that already has been applied to viral ecology (line 80).

However, we do want to highlight important distinctions between the worked referenced here and the work we presented:

1. First, the papers cited focus on viruses infecting the eukaryotic world whereas in our study we focused on viruses infecting bacteria.
2. Second, the paper cited predicts virus-host associations using host phylogeny and geographic overlap as the predictive feature of host range. Although there is great accuracy to predict virus-host associations in those fields, this approach cannot be applied to viruses and microbes.

At this time, we do not think it is appropriate to compare the accuracy of the models cited by reviewer to the model we developed here as they use fundamentally different approaches to predict virus-host associations (i.e., phylogeographic proximity and/or in conjunction with

network-driven models versus DNA-sequence based similarities). While phylogeographic-based approaches might be appropriate and powerful to predict virus-host interactions for species that have a defined spatial arrangement, this type of approach would not work for microbial hosts as they exhibit very weak phylogeographic patterns owing to their relatively unlimited dispersal (Meyer at al 2018).

Nonetheless, the reviewer poses a compelling point that we think we are in deep agreement on. We changed the wording throughout the text to emphasize that our focus is on the study of virus-microbe interactions and the unique challenges of predicting those interactions.

Concerns about the newly assembled dataset:

Collecting lab-verified interactions is an excellent way to train and validate such a model, especially if you have “true negatives”, which are often absent from many databases of host-virus interactions. When presenting your dataset, it would be great to indicate what evidence you considered as proof of a successful infection. For example, if you found the same virus-host interaction tested in two studies, one successful, another not, how would you code this? Would you discard the unsuccessful experiment, or does your model allow for both of these observations as input?

This is a great question. We did not find conflicting results regarding virus-host interactions but we suspect this is because of our approach. We sought to recover lab-tested virus-host interactions associated with the publication of the viral sequence, as we wanted to have the host range of a virus that is best associated with the sequence published.

Your question, however, is a very interesting theoretical challenge. In the hypothetical situation where conflicting host ranges would be reported, we would imagine that this would come from mutations in viral genomes. Hence we focused on retrieving virus-host associations that are the most accurate for the viral genome published.

In addition, we were also interested in reaching out to primary contacts for the viral genomes published to inquire about updated host range, either from re-testing hosts or from challenging new hosts. We sent email to 47 different authors. Out of those, we only heard back from 19 authors. And out of those, only 1 person had an updated host range to share. This was a very time consuming effort that we did not think was worth continuing.

In terms of the provenance of the input data for “VHRnet”, for the NCBI data it is unclear how “clean” or reliable the host tage in GenBank is. Having worked with these data, I have found multiple entires where this information is incorrect. What steps did you take to verify these data? Even manual curation of a small random subset would be a good attempt. Further, if you are using RefSeq, how do you determine if the sequence comes from a lab controll experimental infection rather than an observational study (e.g. virus may be present as a contaminant), and in

this context what does a “non-infection” study look like in terms of the GenBank meta-data? For example, in lines 151-152 you say the majority of viruses were reportedly tested again a single host, and these pairings come from the “host” tag, however the host tag could be the source from which the virus was sampled and a genome was assembled from metagenomic data, but this does not tell you if this was an experimental infection or not.

Thank you for bringing this issue up! There was a mistake in the manuscript. We did not use the “host” tag but the “lab host” tag. The “lab host” tag is only present in virus GenBank files for which a virus was isolated in a laboratory setting. You can find more information at this link: https://www.insdc.org/submitting-standards/feature-table/. We took no step to verify the data as we assumed that the information contained under the “lab host” tag is correct.

We were only able to recover single infection virus-host pairs from GenBank metadata. Those files did not contain non-infection data. All non-infection data collected in VHRnet came from literature research. We hope this clarifies your concerns!

It seems like the majority of your data come from the Nahant study. This database appears to be sampled from one ecosystem (a littoral marine zone). Further, there is no discussion of whether the three sources you used are comparable in terms of the definition of a successful infection, or how this was assessed. Considering over 70% of your virus-host pairs come from this study, and another on Staphylococcus, and you focus extensively on these two datasets in your results, I wonder if it is appropriate to say you have created a new database of lab-tested host-virus interactions.

The word database was not the correct terminology and we changed the wording to dataset. Thank you for pointing it out. Successful infection for viruses infecting bacteria is typically determined by observing clearing of the microbe by the virus in a laboratory setting. For virus-host infection pairs extracted from the GenBank metadata file, we cannot know the method used to determine successful viral infection.

Further, this raises the question of how confident can you be with your predictions as they extend to hosts in other systems (e.g. human microbiome, phyllosphere, soil microbiome, animal microbiomes, etc...)?

This is a great question. This tool could be used as a hypothesis generating tool for following experiments and testing this question, but this is outside the scope of this paper. Moreover, the underlying assumption behind VHIP and existing tools is that viruses have predictable and consistent coevolutionary signals that emerge from genomic sequences. If that assumption is violated for any reason (i.e., viruses from different environments do indeed have different adaptations), then one should be very cautious when interpreting predictions from VHIP or other existing tools that use genomic sequences as the input.

We added a paragraph (line 431) about this in the Discussion section.

Also a small semantic point as mentioned above, this database is actually more of an edge list (list of host-virus interactions, along with their successful experimental infection).

We changed the wording from database to dataset instead.

Line 196-197: You say that existing HPTs performed better in predicting hosts for viruses in the Staph study than viruses in Nahant. Could this be due to an imbalance in your training data? E.g. You have more data for Staph viruses, hence are less able to generalize to other host groups? Considering the potential data quailty and imbalance issues I would have expected some exploration of data re-sampling and/or attempting to fit your model on one dataset and then predict the other.

We used existing tools as advertised. Those tools were trained on known infection virus-host pairs extracted from GenBank files and made assumptions regarding virus host range to generate "non-infection" data (i.e., viruses have narrow host ranges). We did not attempt to retrain those existing tools with the dataset compiled as part of this study as those tools were not designed to be retrained by users but rather be used as is. It is certainly true that those existing tools have seen more viruses infecting the Staphylococcus genus than the Vibrio genus. In addition, because they have a set list of bacterial species they can predict, it is possible that it is missing species relevant to the Nahant Collection, and thus would not be able to predict the corresponding taxa.

We did explore data resampling. The process is outlined in Fig. 1E. The methods for the resampling start on line 576. Because non-infection data points were more prevalent, we downsampled the data to reach a 60 to 40 ratio of non-infection to infection. To determine the best hyperparameters, we ran an exhaustive grid search. And because this process may be sensitive to which data points are used during the learning phase, we bootstrapped 100 times and computed scoring metrics for each bootstraps (Fig. S7 and Fig. S8).

As suggested per your comment, we now also explored resampling from data sources such as the Nahant Collection, the Staph study, and the NCBI host range were represented equally. This reduced the number of data points for training/testing machine learning models from 8849 to 3159. This resulting model has worse score metrics than VHIP (accuracy: 0.829, F1 score: 0.77, ROC: 0.89, and Matthew's correlation: 0.639), which is expected as less data was used for the training phase of the model. This inferior model was then applied on the data unused during the training and it predicted interactions with a 91% accuracy rate. This suggests that data provenance is not a significant driver of the accuracy of VHIP when trained on the entire dataset. We included this new analysis (line 363) in the results section and in the method section (line 590).

Lines 329-336: You should be able to assess whether models are overfit by testing on a hold-out / validation test set. In my experience, models are picked based on performance (assessed via multiple metrics) on validation set, rather than that number of trees. This also limits your ability to compare models which do not have this underlying architecture.

We assessed different machine learning model performance using a hold-out set: 70% of the data was used for training/testing, and the remaining 30% was used for assessing the model performance on data it has not seen during the training phase. We bootstrapped this process for each different type of machine learning model. The results are shown in S6 Fig.

From this analysis, we found that the two best performing models were the Random Forest Classifier and the Gradient Boosting Classifier. We chose the latter as the tree depth required to get a performing model was significantly smaller than the Random Forest classifier (line 410).

Line 336: What is the “untouched dataset”? This should be defined earlier.

By untouched set, we meant the hold out set. We cleared this confusion by changing the wording from “untouched” to hold-out set. Thank you for pointing this out!

Lines 329-361: Much of this reads like Methods rather than results. It would be good to see more results in the context of predictive accuracy across different taxonomic groups, as a function of the input data (which of the data subsets the models were trained on), and discussing some examples of when the model is successful, and then it fails. By examining which interactions your model is predicting as false positives and false negatives you may be able to get some useful insights into why it may fail, and how to build a better next iteration of your model.

Thank you for this point. We carefully considered rearranging the order and location of this information and consulted with outside readers. Our conclusion was that we believe that the paragraph is needed to contextualize the results.

Figure 6: It is unclear how your model extrapolates predictions from the species through to the community and ecosystem levels.

Regarding figure 6, it is an outlook for future research that is made possible by considering all viruses to all microbes for a given microbial community. The tool developed presented here is only one of a number of new approaches that are expanding viral research. Technological advances in sequencing, such as Hi-C, are also allowing this type of analysis. We are currently applying VHIP on new exciting metagenomic data to resolve such virus-host interactions networks across environmental gradients that is outside the scope of this manuscript. However, we want to start the conversation in the literature about the importance and usefulness of the type of “all against all” networks for virus-microbe interactions that this work and others are paving the way for.

Minor comments

Lines 30-31 & 69-70: Please provide more context for “Advances in genome sequencing have

led to the discovery of millions of novel viruses" – it is unclear which species concepts you are applying here, as there are fewer than 7,000 recognized virus species, and while I am aware of studies using different forms of extrapolation and power laws for estimating millions of viruses, these are quite variable and somewhat contested…

Here we are referencing the number of novel viral populations (or "viral OTUs", vOTUs) reconstructed from metagenomic data and identified as novel based on their degree of sequence similarity to known viruses. vOTUs are defined as viral contigs >10kb where >70% of the shared genes have >95% average nucleotide identity across its members (Gregory et al., 2019). As such, the reference to millions of viruses discovered is not based on extrapolation, but rather on these novel (though also uncharacterized and uncultured) viruses identified via metagenomics sequencing. For further reference, please see https://pubmed.ncbi.nlm.nih.gov/36399502/, which describes the JGI IMG viral sequence database, the most up to date compilation of viral sequences (Camargo et al., 2023): https://img.jgi.doe.gov/cgi-bin/vr/main.cgi, where they report "Here, we present the fourth version of IMG/VR, composed of >15 million virus genomes and genome fragments, a ≈6-fold increase in size compared to the previous version. These clustered into 8.7 million viral operational taxonomic units, including 231 408 with at least one high-quality representative."

We have included additional citations to this point.

Lines 32 & 34 & 79: Stylistic comment, but I think "what" is more appropriate than "who" when talking about bacteria and viruses.

We appreciate your point of view and we think that either what or who can be appropriate when talking about bacteria and viruses. Because we are framing the research question of "who infects whom?" we will keep using "who", as it matches the colloquial style that references this grand challenge. For instance, here are some papers that uses "who/whom" for virus/host references:

- https://www.nature.com/articles/s41579-021-00530-x
- https://www.nature.com/articles/nrmicro3404
- https://www.ncbi.nlm.nih.gov/pmc/articles/PMC5352818/
- https://academic.oup.com/bioinformatics/article/33/19/3113/3964377
- https://www.nature.com/articles/s41396-019-0580-z

Line 38: "host range" is often used when discussing the diversity of hosts a pathogen can infect (e.g. how many hosts / species richness, or perhaps phylogenetic diversity, etc.), as a reference to specialism and generalism in terms of host tropism, rather than the particular host-pathogen interaction. Unless you are explicitly predicting host range, using this phrase is incorrect.

For viruses infecting bacteria, the term host range can have two meanings. This term has been used to describe viruses that can infect many different bacterial strains belonging to the same species in order to differentiate with viruses that are only able to infect 1 or few strains. The other term definition is akin to what you mentioned regarding host tropism. On line 38, we are using the former definition of host range.

Line 40: Unclear what “features of co-evolution” are.

We changed the wording “features of co-evolution” to “meaningful signals of viral adaptation to host” (line 40).

Lines 45 & 76: Unlear what you mean by “popuation genomes”. Please define this and differentiate from genomes typically associated with individual species.

The term “population genome” is the term commonly used to describe genomes reconstructed from metagenomic datasets—as referenced, e.g., in the abstract ([line 45] “...can be applied to novel viral and host population genomes reconstructed from metagenomic datasets”). The phrasing distinguishes the composite genome sequence derived from many individuals of a population (as is the case when metagenomic reads from many individuals are assembled into a “genome” sequence). This is different from the case of isolating a clonal virus in the lab and sequencing the genome of the clone. This is important in our study because VHIP accepts viral and microbial host genomes as inputs—which means it can be used to predict virus-host pairs using “novel viral and host population genomes reconstructed from metagenomic datasets” (from the abstract).

Line 82: Is “host prediction tools (HPT)” a commonly used term in this field? I am more familiar with host-virus link prediction models / network models, etc...

Host prediction tool (HPT) is a general term we define and use here to describe the tools we referenced in our study. The use of HPT was a stylistic shorthand to help with brevity and ease of reading. We aren’t sure if this is a suggestion for using a different term so will err on the side of thoroughness. In considering this comment, we concluded that host-virus link prediction model is not an appropriate term for the tools we compared in this manuscript. This is because those tools focus on predicting the most likely taxa a virus may infect. They do not predict individual virus-host links, nor can they be used to resolve interaction networks. To differentiate between this existing approach and our approach to consider all possible virus-host pairs, we felt it was necessary to emphasize the difference, hence “host prediction tools”.

Line 91-93: I don’t understand the idea that these models “do not predict non-infection”. Aren’t most of these models assuming a binary outcome, and thus predicting an interaction for some host-virus paris also by necessity predicting the absence of a similar interaction in a different

host-virus pair? If HPTs are very different than existing network prediction / species interaction / link prediction models, it is important to offer a more detailed summary of these approaches and how they differ.

The tools mentioned in this study take input viruses and assess those viruses against a predefined database of bacterial hosts. Those tools typically return a single or multiple potential hosts. However, those tools assess their accuracy only on their best (i.e., highest score) prediction. To illustrate this point, here is an excerpt from the most recent tool published, IPHoP, which also compared the tool: “For all the tools, the best prediction was taken for each input virus based on the relevant metric and considered as correct if the genus of the predicted host genome or the predicted genus for tools predicting host taxonomy was consistent with the information collected from the reference database”.

We did not offer a detailed summary of these approaches as this was deeply explored in a review which is cited in this manuscript: Coclet C, Roux S. Global overview and major challenges of host prediction methods for uncultivated phages. Curr Opin Virol. 2021 Aug 1;49:117–26.

Lines 101-103: Given the format of results then methods, it would greatly help the reader if you have a breif overview of the types of co-evolutionary signals you measured (are these identified sites of selection, or auto-generated genome composition features?), and the general type of ML model employed (GBM) somewhere here in the introduction.

Figure 1 outlines the order by which we introduce the data collected, the virus-host coevolution signals considered, and the machine learning model approach. We do not feel like it is necessary to expand on the signals of coevolution and machine learning model approaches in the introduction as it has been the primary focus of existing host prediction tools. Our aim with this manuscript is to emphasize the benefits of incorporating both infection and non-infection data points that we collected from literature.

Lines 110-112: Please clarify that these are bacteriophages and bacteria, correct? If so, these are not all viruses and hosts as you exclude large taxonomic groups of viral hosts here.

Yes, this study focuses on viruses infecting bacteria or archaea. This study excludes any viruses that infect eukaryotic groups. We avoid using the word bacteriophage because 1) it excludes viruses infecting archaea, and 2) has been routinely used in our subfield studying viruses of microbes. We changed the text to emphasize that the hosts are microbial (bacteria or archaea).

Line 308-312: What cutoff for correlation did you use? Depending on the ML model used, some relatively “highly correlated” predictors could provide very useful information. For example, in GBMs, which allow for highly non-linear interactions between predictors, predictors which

appear fairly correllated could actually provide important discriminatory information for particular classes.
Only two features that we considered were highly correlated (Pearson = 0.94). We opted to keep both of them (line 305).

Line 352: You first use AUROC, then here switch to ROC when referring to AUROC.

Thank you for catching this mistake. This has been fixed.
