# Supplementary material for: Virus-host interactions predictor (VHIP): Machine learning approach to resolve microbial virus-host interaction networks
Source: PLoS Comput Biol. 2024 Sep 18;20(9):e1011649. doi: 10.1371/journal.pcbi.1011649 (PMC11441702; doi:10.1371/journal.pcbi.1011649)
Supplement: Attachment — Submitted filename: response_to_reviewers.pdf [file pcbi.1011649.s015.pdf]

Response to reviewers

Dear Dr. Duhaime,

Thank you very much for submitting your manuscript "Virus-Host Interactions Predictor (VHIP): machine learning approach to resolve microbial virus-host interaction networks" for consideration at PLOS Computational Biology. As with all papers reviewed by the journal, your manuscript was reviewed by members of the editorial board and by several independent reviewers. The reviewers appreciated the attention to an important topic. Based on the reviews, we are likely to accept this manuscript for publication, providing that you modify the manuscript according to the review recommendations.

I agree with both reviewers that the manuscript has improved substantially during revision. I would ask the authors to provide the comparisons requested by reviewer 2 and to ensure that the points raised by reviewer 3 in their original assessment are discussed in the manuscript (in addition to the response letter). I leave the decision around whether to keep figure 6, move it to the supplement, or save it for a future paper to the authors. However, I agree with the sentiment of reviewer 2 that the figure doesn't add much and is pretty high-level.

Please prepare and submit your revised manuscript within 30 days. If you anticipate any delay, please let us know the expected resubmission date by replying to this email.

When you are ready to resubmit, please upload the following:

[1] A letter containing a detailed list of your responses to all review comments, and a description of the changes you have made in the manuscript. Please note while forming your response, if your article is accepted, you may have the opportunity to make the peer review history publicly available. The record will include editor decision letters (with reviews) and your responses to reviewer comments. If eligible, we will contact you to opt in or out

[2] Two versions of the revised manuscript: one with either highlights or tracked changes denoting where the text has been changed; the other a clean version (uploaded as the manuscript file).

Important additional instructions are given below your reviewer comments.

Thank you again for your submission to our journal. We hope that our editorial process has been constructive so far, and we welcome your feedback at any time. Please don't hesitate to contact us if you have any questions or comments.

Sincerely,

Samuel V. Scarpino

Academic Editor

PLOS Computational Biology

James O'Dwyer

Section Editor

PLOS Computational Biology

****

A link appears below if there are any accompanying review attachments. If you believe any reviews to be missing, please contact ploscompbiol@plos.org immediately:

I agree with both reviewers that the manuscript has improved substantially during revision. I would ask the authors to provide the comparisons requested by reviewer 2 and to ensure that the points raised by reviewer 3 in their original assessment are discussed in the manuscript (in addition to the response letter). I leave the decision around whether to keep figure 6, move it to the supplement, or save it for a future paper to the authors. However, I agree with the sentiment of reviewer 2 that the figure doesn't add much and is pretty high-level.

Thank you again for taking the time and effort to review our manuscript. We addressed the reviewers' comments as best as we could and adapted the manuscript to address reviewer feedback.

Reviewer's Responses to Questions

Comments to the Authors:
Please note here if the review is uploaded as an attachment.

Reviewer #2: Thank you for your response to my comments and the changes you have made in the revision. I do have two further comments regarding to your response.

To your response to my 2nd major comment:
I think the author can still compare the accuracy of VHIP with existing tools 1) based on the conventional metric (e.g. either prediction of species A or species B is considered 100% accurate), and 2) based a new metric (e.g. only prediction of both species A and B is considered 100% accurate). In the latter case, some existing tools may still be applicable since they can make multi-host prediction based on a threshold cutoff of confidence score. I think both metrics have applicable scenarios in virus-host studies.

We thank the reviewer for continuing to tackle this challenge that we have grappled with for months. The first approach suggested here indeed would suffice for tools that are designed to predict correct taxa, but would unfortunately inflate the accuracy for VHIP. While that would be nice to show the superiority of our method, we believe it does not present a fair objective comparison. This is because the suggested approach evaluates the potential of a tool to predict

at least one correct host, while ignoring all wrong predictions. For instance, using the example given by the reviewer, if VHIP correctly predicted species A and incorrectly predicted species B, the true accuracy is 50%. The accuracy based on the metric proposed here would result in 100% accuracy since it predicted at least one host correctly. We do not feel comfortable applying this definition of accuracy as it might mislead users and suggest VHIP is more accurate than it truly is.

However, the second approach suggested has been implemented. We added a paragraph on line 377 (starting with “Comparing VHIP to existing host prediction tools is not straightforward”) in the results section where we compare VHIP and existing tools in their ability to recover the known host range of viruses. The accuracy using this metric is reported on line 387.

To your response to my 4th major comment:
I still think Fig. 6 is immature and it breaks the integrity of this manuscript. Please save it for your next paper.

Indeed, Figure 6 has received mixed feedback. We truly appreciate the sentiments of this reviewer and their candid reaction. We certainly considered removing it upon receiving this feedback, but reconsidered given the positive response we also receive when sharing this graphical overview of the project and the vision for its future applications. In line with the editor’s support to include it at our own discretion, we will leave in a modified version. We find the figure to be, at worst, a net neutral inclusion, rather than net negative, given that there is nothing incorrect, unethical, or inappropriate, in terms of data representation or scientific integrity. The offense to the figure, when one is stated, seems to be a stylistic offense. Whereas when it is appreciated, it serves to effectively communicate the fundamental principles of the project in a graphical way that appeases visual communication and learning styles.

Reviewer #3: I reviewed a previous version of this manuscript. The authors have addressed some of my concerns, including exploring potentially biased data through sub-sampling, and stating why some classes of link prediction models developed for vertebrate host-virusses (e.g. phylogeographic models) may not be applicable to predict microbe-virus interactions.

For my other concerns, I find that the authors address these in the response letter but it is unclear that they have made appropriate changes in the manuscript (e.g. viral species concepts / vOTUS are not mentioned, “features of co-evolution” are still mentioned and genomic features are not explicitly linked to co-evolutionary mechanisms, and it is unclear why HPTs are different than host-virus link prediction models).

We thank the review for their astute attention to these details. We have modified the latest version of the manuscript in the following ways:

Response for viral species concepts. From the first round of reviews, we indeed had included additional citations in our resubmission (lines 67, citations: Gregory 2019, Roux 2017, Camargo 2023). We now more clearly understand that the reviewer was expecting the topic to be expanded upon more explicitly in the text. We now adapted the introduction as such ("Those viral populations have been reconstructed from metagenomic data and identified as novel based on their lack of sequence similarity to known viruses, expanding the total number of distinct uncultured viruses to millions in the last decade alone").

Response for features of coevolution. We agree with the point raised by this reviewer and appreciate the concept being brought to our attention at this stage. In response to this feedback, we have removed the term "features of coevolution" completely from the manuscript and changed it to the term "genomic signal". We also better defined the origin of those genomic signals (lines 86: "This approach of leveraging genomic signals to predict virus-host association is possible because viruses rely on their host machinery to complete their life cycle and evolve to better utilize those resources by matching the codon biases of their host. This results in a meaningful and capturable signal that is embedded in the sequences of the viruses and their known host."). We reserve the term "features" to exclusively refer to data components used to train and test machine learning models, as is customary for these model types; in our case, the term "features" is used to refer to the quantitative descriptors of virus-host pairs (e.g., GC difference, k-mer profile distances) that serve as model inputs. Thank you for pointing out this semantic and technical inconsistency and we hope this addresses your concern.

Response for linking genomic features and coevolutionary mechanisms. Thank you for your feedback in pointing out this gap. We expanded the introduction to more explicitly describe the logic behind the evolutionary mechanisms and the resulting genomic features in line 86 ("This approach of leveraging genomic signals to predict virus-host association is possible because viruses rely on their host machinery to complete their life cycle and thus evolve to better utilize those resources by matching the codon biases of their host.This results in a capturable signal that is embedded in the sequences of the viruses and their known hosts")

Response to why HPTs are different from host-virus link prediction models. We appreciate the attention brought to the host-virus link prediction models that can be better understood and communicated through the lens of host-virus link prediction models. The function of HPTs is described in the introduction on line 84 ("Another method leverages patterns of coevolution that can be extracted from the genomic sequences of the viruses and their host to predict the most likely taxa that can be infected by a given virus"). This limitation of only predicting taxa is mentioned again in the Results section on line 193 ("by HPTs (that is, only one known host per virus), these tools typically deliver only their highest scoring host prediction as output". Since the focus of those tools is to predict the correct taxonomic assignment of a host, we felt it was necessary to emphasize the difference, hence "host prediction tools". In contrast, if we were to use the term "host-virus link prediction models" to refer to those existing tools, it would suggest that those tools could predict multiple links and that both viruses and hosts are inputs of those tools. Since this is not the case, we opted to use the term HPT which better reflects the technical design of those tools.
